# Marine algal (seaweed) flora of Terceira Island, Azores

**DOI:** 10.3897/BDJ.8.e57462

**Published:** 2020-10-02

**Authors:** Ana I. Azevedo Neto, Afonso C. L. Prestes, Nuno Vaz Álvaro, Roberto Resendes, Raul M. A. Neto, Ignacio Moreu

**Affiliations:** 1 cE3c - Centre for Ecology, Evolution and Environmental Changes/Azorean Biodiversity Group & Faculdade de Ciências e Tecnologia, Departamento de Biologia, Universidade dos Açores, 9500-321 Ponta Delgada, São Miguel, Açores, Portugal cE3c - Centre for Ecology, Evolution and Environmental Changes/Azorean Biodiversity Group & Faculdade de Ciências e Tecnologia, Departamento de Biologia, Universidade dos Açores 9500-321 Ponta Delgada, São Miguel, Açores Portugal; 2 Universidade dos Açores, Faculdade de Ciências Agrárias, CCMMG (Centro do Clima Meteorologia e Mudanças Globais), IITA-A (Instituto de Investigação e Tecnologias Agrárias e do Ambiente), 9700-042 Angra dp Heroísmo, Terceira, Portugal Universidade dos Açores, Faculdade de Ciências Agrárias, CCMMG (Centro do Clima Meteorologia e Mudanças Globais), IITA-A (Instituto de Investigação e Tecnologias Agrárias e do Ambiente) 9700-042 Angra dp Heroísmo, Terceira Portugal; 3 Universidade dos Açores, Faculdade de Ciências e Tecnologia, Departamento de Biologia, 9500-321 Ponta Delgada, São Miguel, Açores, Portugal Universidade dos Açores, Faculdade de Ciências e Tecnologia, Departamento de Biologia 9500-321 Ponta Delgada, São Miguel, Açores Portugal; 4 N/A, Odivelas, Portugal N/A Odivelas Portugal

**Keywords:** Macroalgae, seaweeds, Rhodophyta, Chlorophyta, Ochrophyta, Azores, Terceira Island, endemism, native, introduced, uncertain, occurrence data

## Abstract

**Background:**

As for many other Azorean Islands, the macroalgal flora of Terceira (belonging to the central group of the archipelago) is poorly known, the published information reflecting occasional collections of sporadic visitors to the island. In order to overcome this and contribute to improve the knowledge of Azorean macroalgal flora at both local and regional scales, a thorough investigation was conducted. Both collections and presence data recordings were undertaken at the littoral and sublittoral levels down to approximately 40 m around the island, covering a total area of approximately 49 km^2^. This paper lists the taxonomic records and provides information on each species’ ecology and occurrence on the Island’s littoral.

**New information:**

A total of 418 specimens (including taxa identified only to genus level) belonging to 147 taxa of macroalgae, comprising 95 Rhodophyta, 33 Chlorophyta and 19 Ochrophyta (Phaeophyceae) are registered. Of these, 113 were identified to species level (73 Rhodophyta, 24 Chlorophyta and 16 Ochrophyta), encompassing 35 new records for the Island (27 Rhodophyta, 6 Chlorophyta and 2 Ochrophyta). Most species are native, including the Macaronesian endemisms *Codium elisabethiae* O.C.Schmidt, *Millerella tinerfensis* (Seoane-Camba) S.M.Boo & J.M.Rico and *Phyllophora gelidioides* P.Crouan & H.Crouan ex Karsakoff. Eight species are introduced and 15 have uncertain origin.

## Introduction

The macroalgal flora of the isolated mid-Atlantic Azores archipelago, as a whole, may be considered relatively rich when compared to that of other remote oceanic islands, such as the Shetlands and Faroes in the colder North Atlantic and Ascension and Tristan da Cunha in the Southern Atlantic (Neto et al. 2005, Tittley and Neto 2005, Wallenstein et al. 2009). With approximately 400 species (Freitas et al. 2019), the Azorean algal flora has been considered cosmopolitan, as it shares species with Macaronesia, North Africa, the Mediterranean Sea, Atlantic Europe and America (Tittley 2003, Tittley and Neto 2006, Wallenstein et al. 2009).

The published information, however, reflects data from only a few of the nine islands. Terceira, the second largest island of the central group and the third largest of the archipelago, is amongst the lesser-studied ones. To overcome this and contribute to a better understanding of the seaweed flora of the Azores archipelago, a thorough investigation was conducted in the period between 2000 and 2014, mainly by the Island Aquatic Research Group of the Azorean Biodiversity Centre of the University of the Azores (https://ce3c.ciencias.ulisboa.pt/sub-team/island-aquatic-ecology). In these surveys, special attention was dedicated to the sheet-like and filamentous forms that are difficult to identify in the wild, the seasonal and fast growing annuals and particularly to the small forms that are often short-lived and fast growing species, very difficult to identify without the aid of a microscope. This paper compiles physical, occurrence and survey data and is intended as a practical resource for biological studies (such as systematics, diversity and conservation, biological monitoring, climate change and ecology) and for academics, students, government, private organisations and the general public.

## General description

### Purpose

By listing the taxonomic records for Terceira and presenting general information for each taxon’s occurrence on the Island’s littoral, this paper addresses several biodiversity shortfalls (see Cardoso et al. 2011, Hortal et al. 2015), namely the need to catalogue the Azorean macroalgae (Linnean shortfall) and improve the current information on their local and regional geographic distribution (Wallacean shortfall), as well as on species’ abundances and dynamics in space (Prestonian shortfall).

## Project description

### Title

Marine algal (seaweed) flora of Terceira Island, Azores

### Personnel

Collections were undertaken and occurrence data recorded during several years (2000-2014) under the coordination of Ana I. Neto. Main collectors were Afonso Prestes, Albert Cámara, Ana I. Neto, Luís Cabral, Mariana Brito, Marisa Toste, Marlene Terra, Nuno Álvaro and Rita Patarra. Ana I. Neto and Marlene Terra were responsible for the species identification.

Voucher specimen management was mainly undertaken by Afonso Prestes, Ana I. Neto, Eunice Nogueira, Natália Cabral and Roberto Resendes.

### Study area description

**Figure 1.**
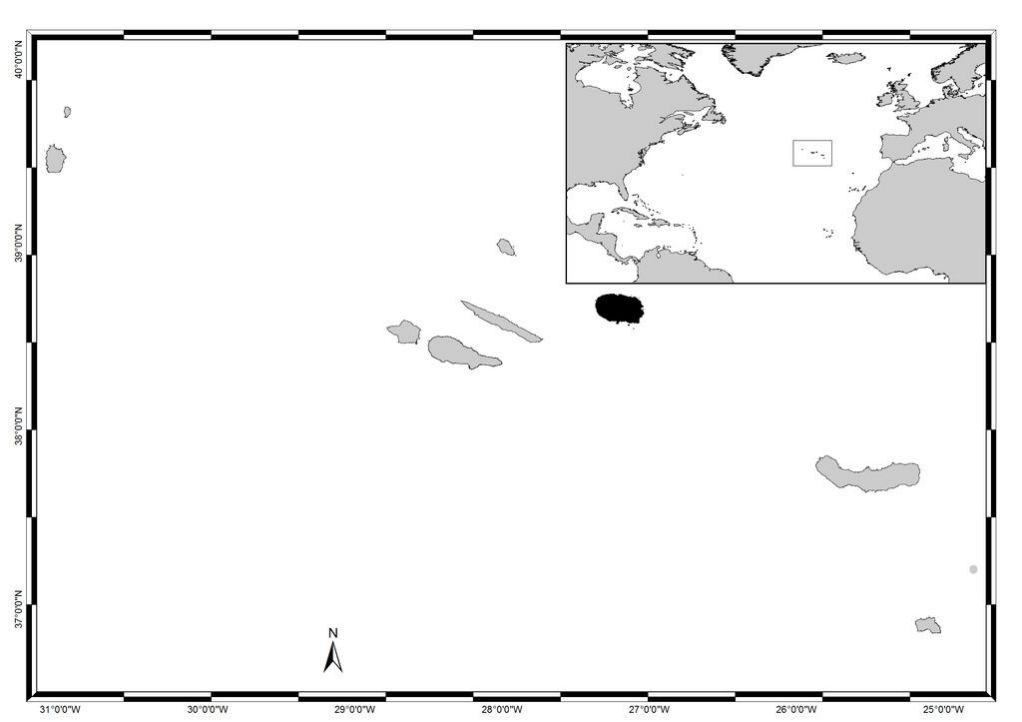
The Azores, its location in the Atlantic and Terceira Island highlighted in black (by Nuno V. Álvaro).

Located along a WNW-trending strip and spreading over 500 km in the North Atlantic, roughly at 38°43′49″N, 27°19′10″W (Fig. 1), the Azores archipelago is composed of nine islands and several islets. The islands are surrounded by deep waters due to the absence of a continental shelf and, therefore, have a restricted coastal extension, which is subjected to swell and surge most of the year. The tidal range is small (< 2 m, see Hidrográfico 1981) and the shore geomorphology alternates between high cliffs and rocky cobble/boulder beaches (Borges 2004). The climate is temperate oceanic, with regular and abundant rainfall and high levels of relative humidity and persistent winds, mainly during winter and autumn (Morton et al. 1998).

Terceira (in black in Fig. 1), located in the central group roughly at 38°48′50″N, 27°23′25″W, 150 km northeast of São Miguel, is the third largest island of the Azores archipelago. It has an elliptical form, 29 km long and 18 km wide, a maximum altitude of 1021 m at the summit of Serra de Santa Bárbara and a total area of about 397 km^2^. The coastline has a total length of 112 km and is characterised by cliffs that vary from small to moderate heights, interrupted by small bays. Sandy beaches are limited to Praia da Vitória, located on the more protected eastern part of the Island. The northern coast is more exposed and constantly submitted to the wave action (Gomes and Pinto 2004).

**Figure 2.**
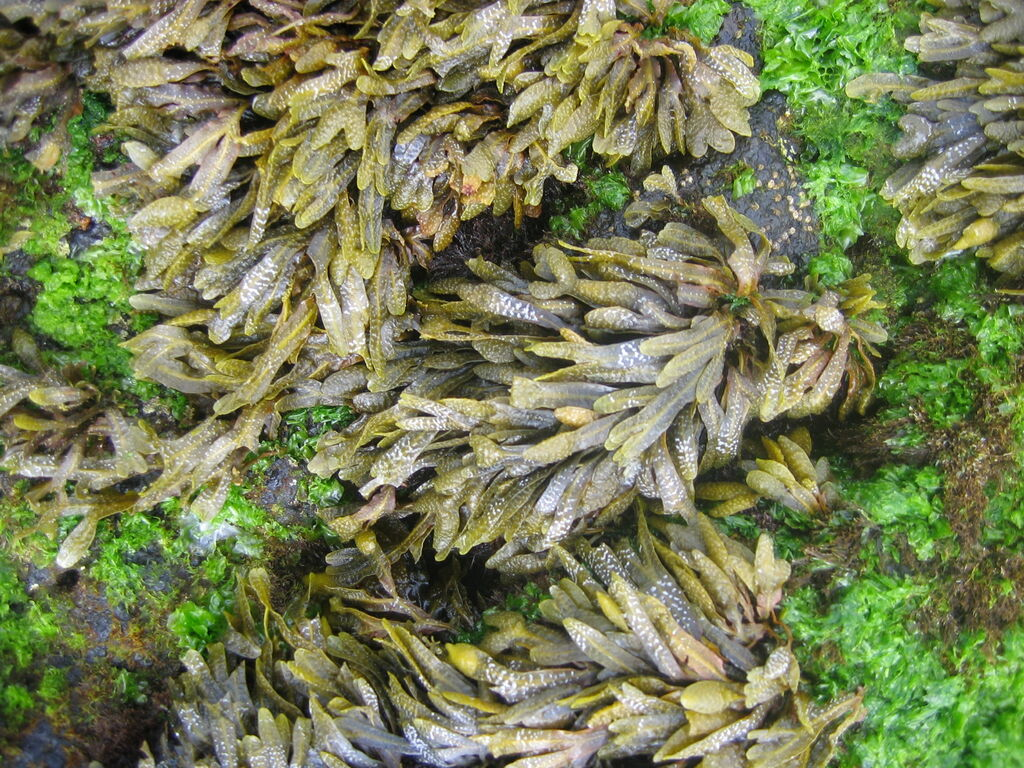
High intertidal level showing *Fucus spiralis* and *Ulva rigida* (by the Island Aquatic Ecology Subgroup of cE3c-ABG).

**Figure 3.**
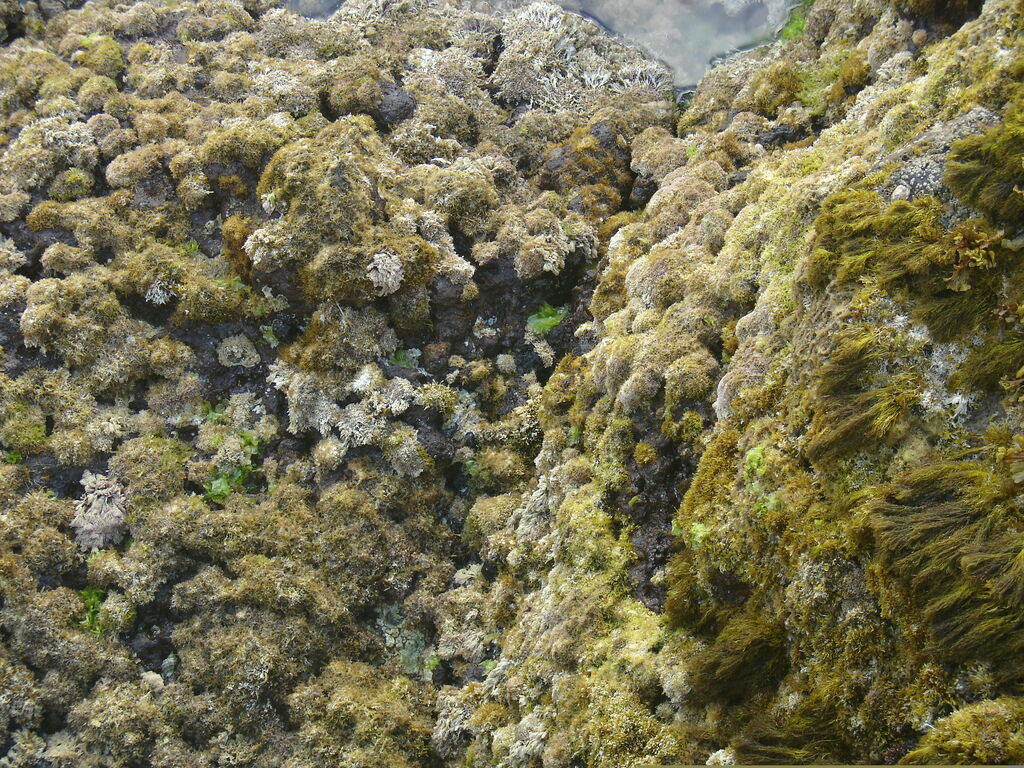
Mid-shore intertidal covered by algal turf (by the Island Aquatic Ecology Subgroup of cE3c-ABG).

**Figure 4.**
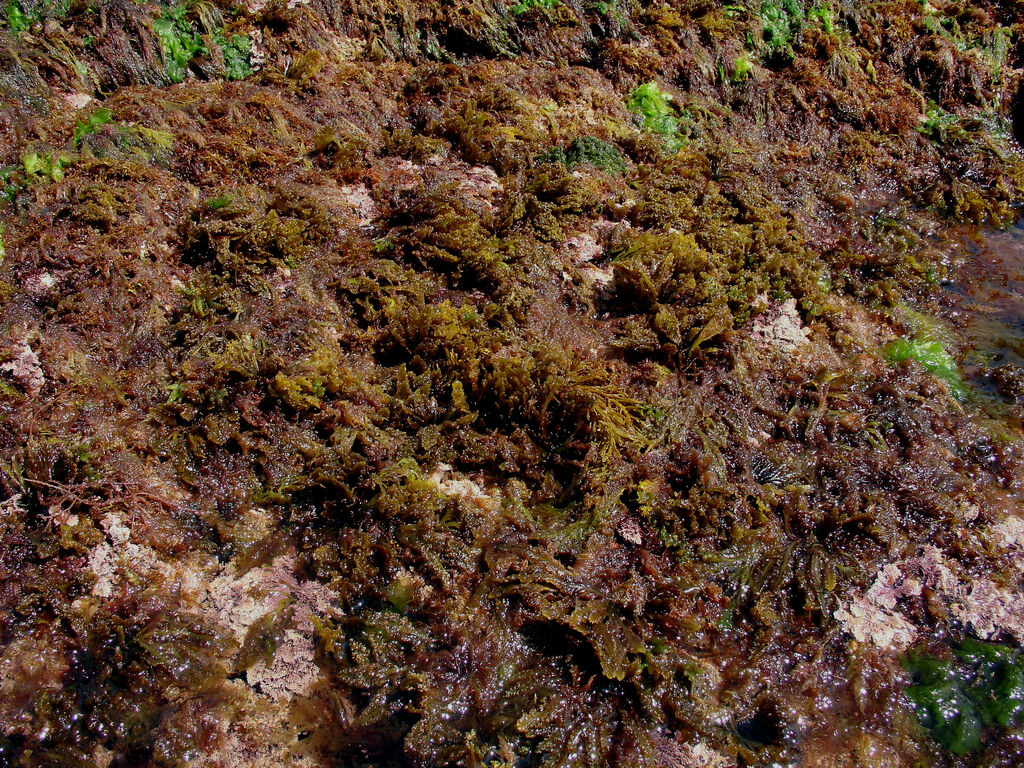
*Cystoseira* sp., *Ellisolandia elongata* and *Osmundea pinnatifida* epiphyting multi-specific algal turf at low intertidal (by the Island Aquatic Ecology Subgroup of cE3c-ABG).

**Figure 5.**
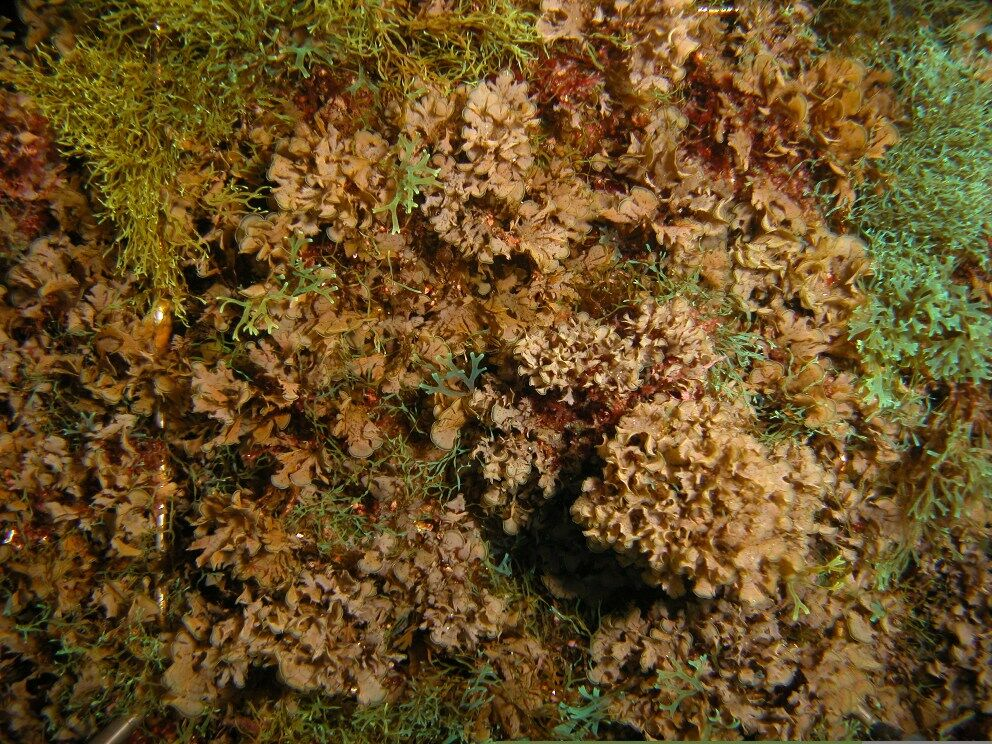
Frondose macrophytes (*Dictyota* spp. and *Zonaria tournefortii*) at subtidal level (by the Island Aquatic Ecology Subgroup of cE3c-ABG).

The intertidal and shallow subtidal rocky-shore communities of Terceira are dominated by macroalgae, similarly to those of the remaining Azorean Islands (Neto et al. 2005). The frondose species *Fucus spiralis* Linnaeus (Fig. 2), *Ulva rigida* C.Agardh and *Gelidium microdon* Kützing are often present at mid-shore levels, growing interspaced with the small chthamalid barnacles. Slightly below this level, the lack of herbivores, resulting from the over-exploitation of limpets (Martins et al. 2011, Martins et al. 2008, Faria et al. 2014), favours an almost homogeneous coverage of the shore by algal turfs (Fig. 3). These are growth forms of either diminutive algae or diminutive forms of larger species that create a dense, compact mat 20-30 mm thick, either monospecific (mainly composed of *Caulacanthus ustulatus* (Mertens ex Turner) Kützing or *Gymnogongrus* spp.) or multi-specific and composed of articulate calcareous algae (e.g. *Ellisolandia elongata* (J.Ellis & Solander) K.R.Hind & G.W.Saunders and *Jania* spp.) and/or soft algae (e.g. *Centroceras clavulatum* (C.Agardh) Montagne, *Chondracanthus* spp. and *Laurencia* spp.). Lower on the shore, the erect, corticated macrophytes *Ellisolandia elongata*, *Cystoseira* spp. and *Osmundea pinnatifida* (Hudson) Stackhouse are commonly found, frequently epiphyting multi-specific algal turfs (Fig. 4). The shallow subtidal is mainly characterised by associations of two or three frondose macrophytes, predominantly the brown seaweeds *Dictyota* spp. and *Zonaria tournefortii* (J.V. Lamouroux) Montagne (Fig. 5).

### Design description

The algae, referred to in this paper, were sampled during field studies at littoral and sublittoral levels down to approximately 40 m on Terceira Island, covering an area of 49 km^2^. Presence recordings and physical collections were made by walking over the shores or by scuba diving. The specimens collected were taken to the laboratory for standard procedures and the resulting vouchers were deposited at the AZB Herbarium Ruy Telles Palhinha, at the Faculty of Sciences and Technology of the University of the Azores.

### Funding

This study was mainly financed by the following projects/scientific expeditions:

- Campaign CAMAG-TER/2008, under the project “CAMAG/TER - Caracterização das massas de água costeira da Ilha Terceira”. 2008 - 2009. The Azores Regional Government;
- Project “ACORES-01-0145-FEDER-000072 - AZORES BIOPORTAL – PORBIOTA. Operational Programme Azores 2020 (85% ERDF and 15% regional funds);
- Portuguese National Funds, through FCT – Fundação para a Ciência e a Tecnologia, within the projects UID/BIA/00329/2013, 2015 - 2018, and UID/BIA/00329/2019 and UID/BIA/00329/2020-2023;
- Portuguese Regional Funds, through DRCT – Direção Regional da Ciência e Tecnologia, within several projects, since 2013;
- CIRN/DB/UAc (Research Centre for Natural Resources, Universidade dos Açores, Departamento de Biologia);
- CIIMAR (Interdisciplinary Centre of Marine and Environmental Research, Porto, Portugal).

## Sampling methods

### Study extent

**Figure 6.**
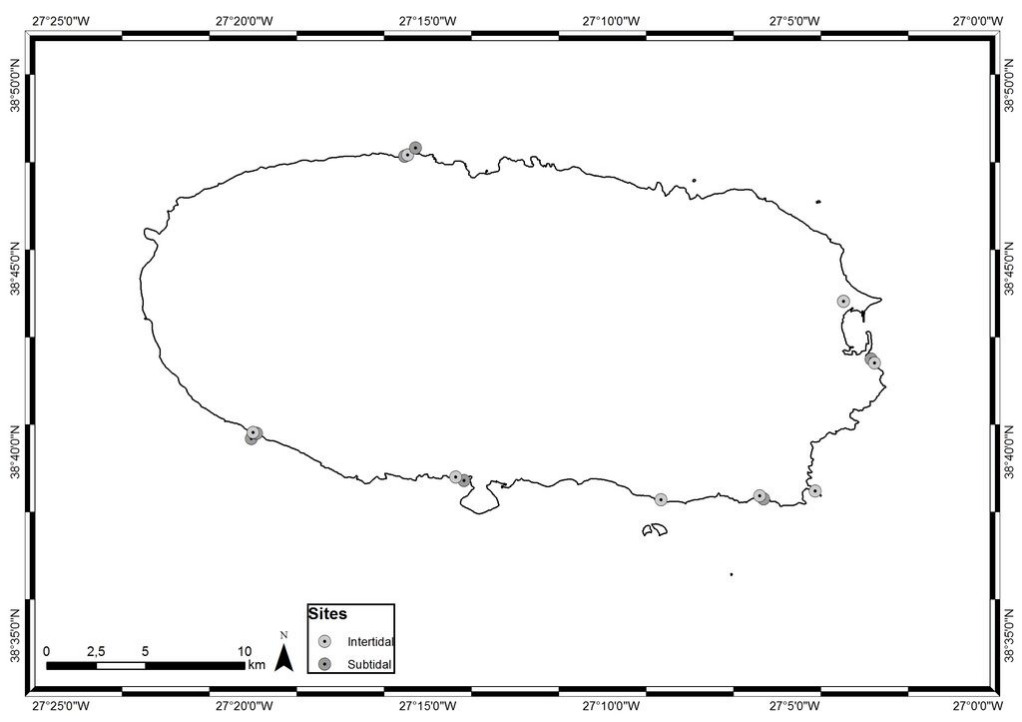
Sampling locations around Terceira Island (by Nuno V. Álvaro).

This study covers an area of approximately 49 km^2^, encompassing littoral and sublittoral levels down to approximately 40 m around Terceira Island (Table 1, Fig. 6).

### Sampling description

**Figure 7.**
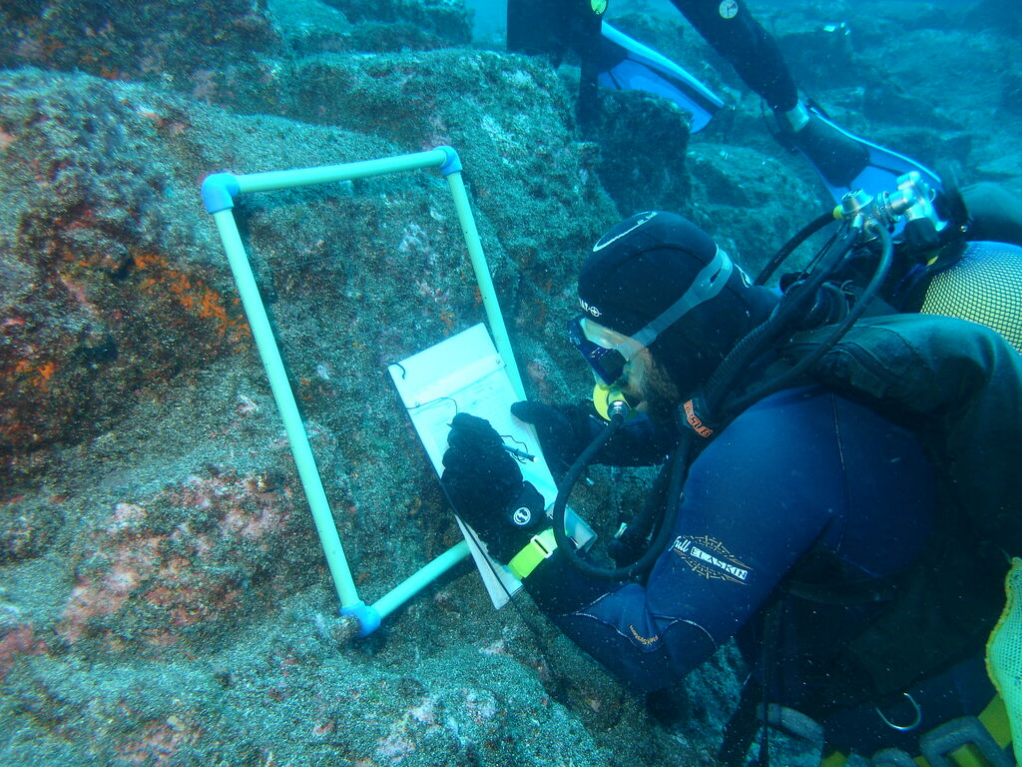
Macroalgae recordings at the rocky subtidal (by the Island Aquatic Ecology Subgroup of cE3c-ABG) .

Intertidal collections were made at low tide by walking over the shores. Subtidal collections were made by scuba diving around the area. Sampling encompassed both physical collections and species presence recordings. For the former, in each sampling location, collections were made manually by scraping one or two specimens of species found into labelled bags. Species recording data were gathered by registering all species present in the visited locations (Fig. 7).

### Quality control

The collected taxa were investigated by trained taxonomists with the help of keys and floras. This involved morphological and anatomical examination by eye or under the dissecting and compound microscopes of an entire specimen or slide preparation. In difficult cases, specimens were sent to experts for identification.

### Step description

In the laboratory, the specimens were sorted and studied following standard procedures used in macroalgae identification.

**Figure 8.**
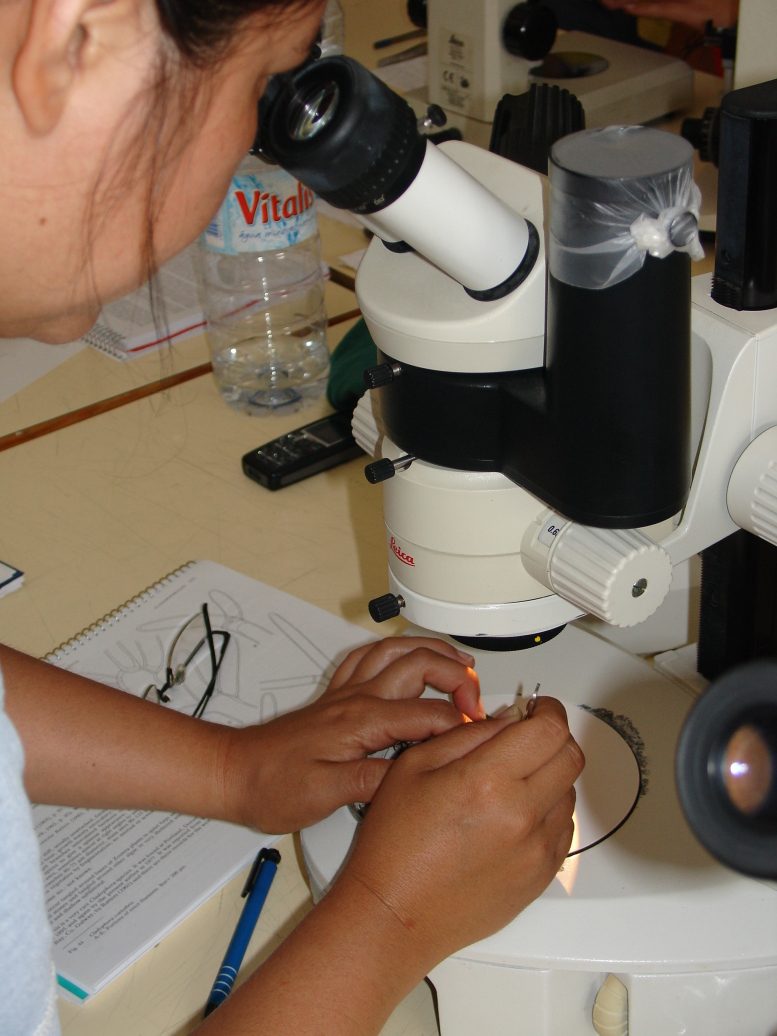
Macroalgae species identification (by the Island Aquatic Ecology Subgroup of cE3c-ABG).

Species identification was based on morphological and anatomical characters and reproductive structures. For small and simple thalli, this required observation of the entire thallus by eye and/or using dissecting and compound microscopes (Fig. 8). For larger and more complex algae, the investigation of the thallus anatomy required histological work to obtain longitudinal and transverse sections needed for the observation of cells, reproductive structures and other diagnosing characters.

Since the Azorean algal flora is composed of taxa from various geographical regions, floras and keys mainly from the Atlantic and Western Mediterranean were used in species identification (e.g. Schmidt 1931, Taylor 1967, Taylor 1978, Levring 1974, Dixon and Irvine 1977, Lawson and John 1982, Irvine 1983, Gayral and Cosson 1986, Fletcher 1987, Afonso-Carrillo and Sansón 1989, Burrows 1991, Boudouresque et al. 1992, Cabioc'h et al. 1992, Maggs and Hommersand 1993, Irvine and Chamberlain 1994, Brodie et al. 2007, Lloréns et al. 2012, Rodríguez-Prieto et al. 2013).

For more critical and taxonomically-difficult taxa, specimens were taken to the Natural History Museum (London) for comparison with collections there or sent to specialists.

A reference collection was made for all specimens collected by giving them a herbarium code number and depositing them at the AZB Herbarium Ruy Telles Palhinha, University of Azores. Depending on the species and on planned further research, different types of collections were made, namely (i) liquid collections using 5% buffered formaldehyde seawater and then replacing it by the fixing agent Kew (Bridsen and Forman 1999); (ii) dried collections, either by pressing the algae (most species) as described by Gayral and Cosson (1986) or by letting them air-dry (calcareous species) and (iii) silica collections for molecular studies.

Nomenclatural and taxonomic status used here follow *Algaebase* (Guiry and Guiry 2020). The database was organised on FileMaker Pro.

## Geographic coverage

### Description

Terceira Island, Azores, Macaronesia, Portugal (approximately 38°48′50″N, 27°23′25″W).

### Coordinates

38.627 and 38.814 Latitude; -27.389 and -27033 Longitude.

## Taxonomic coverage

### Description

All macroalgae were identified to genus or species level. In total, 147 taxa were identified belonging to 21 orders and 45 families, distributed by the phyla Rhodophyta (9 orders and 25 families), Chlorophyta (5 orders and 8 families) and Ochrophyta (7 orders and 12 families).

### Taxa included

**Table:** 

Rank	Scientific Name	Common Name
phylum	Rhodophyta	Red algae
phylum	Chlorophyta	Green algae
phylum	Ochrophyta	Brown algae

## Temporal coverage

### Notes

Sampling took place in the period between 2000 and 2014.

## Collection data

### Collection name

AZB | Marine macroalgae collection of Terceira Island (Azores) – Campaign CAMAG-TER/2008; AZB | Marine macroalgae collection of Terceira Island (Azores) – Occasional sampling; Marine macroalgae occurrence on Terceira Island (Azores) – Campaign CAMAG-TER/2008.

### Collection identifier

389ac3c6-6c63-4de0-b5fb-bc7cc93d3791; 247417a8-f838-405e-b5ac-82940e866a9a; 43bb7387-0e2f-47ce-a121-ca66a9abcaab.

### Parent collection identifier

AZB Herbarium Ruy Telles Palhinha, Faculty of Sciences and Technology of the University of the Azores; AZB Herbarium Ruy Telles Palhinha, Faculty of Sciences and Technology of the University of the Azores; AZB Herbarium Ruy Telles Palhinha, Faculty of Sciences and Technology of the University of the Azores.

### Specimen preservation method

Air-dry, Dried and pressed; Liquid (Formalin; fixing agent Kew), Silica

### Curatorial unit

AZB Herbarium Ruy Telles Palhinha, Faculty of Sciences and Technology of the University of the Azores

## Usage rights

### Use license

Creative Commons Public Domain Waiver (CC-Zero)

## Data resources

### Data package title

Marine algal (seaweed) flora of Terceira Island, Azores

### Resource link

https://www.gbif.org/dataset/b03dce75-cbc2-457b-8725-33885d766a05

### Alternative identifiers

http://ipt.gbif.pt/ipt/resource?r=terceira_seaweed_flora

### Number of data sets

1

### Data set 1.

#### Data set name

Marine algal (seaweed) flora of Terceira Island, Azores

#### Data format

Darwin Core Archive

#### Number of columns

51

#### Download URL

https://doi.org/10.15468/dl.p6pn6w

#### Data format version

version 1.7

#### Description

This data paper presents physical and occurrence data from macroalgal surveys undertaken on Terceira Island between 2000 and 2014. The dataset submitted to GBIF is structured as a sample event dataset, with two tables: event (as core) and occurrences (Neto et al. 2020). The data in this sampling event resource have been published as a Darwin Core Archive (DwCA), which is a standardised format for sharing biodiversity data as a set of one or more data tables. The core data table contains 18 records (eventID). The extension data table has 418 occurrences. An extension record supplies extra information about a core record. The number of records in each extension data table is illustrated in the IPT link. This IPT archives the data and thus serves as the data repository. The data and resource metadata are available for downloading in the downloads section.

**Data set 1.** 

Column label	Column description
Table of Sampling Events	Table with sampling events data (beginning of table)
eventID	Identifier of the event, unique for the dataset
country	Country of the sampling site
countryCode	Code of the country where the event occurred
stateProvince	Name of the region
island	Name of the island
municipality	Name of the municipality
locality	Name of the locality
locationID	Identifier of the location
decimalLatitude	The geographic latitude of the sampling site
decimalLongitude	The geographic longitude of the sampling site
geodeticDatum	The spatial reference system upon which the geographic coordinates are based
coordinateUncertaintyInMetres	The horizontal distance (in metres) from the given decimalLatitude and decimalLongitude describing the smallest circle containing the whole of the Location
eventDate	Time interval when the event occurred
year	The year of the event
samplingProtocol	Sampling method used during an event
locationRemarks	Zonation level
minimumDepthInMetres	The minimum depth in metres where the specimen was found
maximumDepthInMetres	The maximum depth in metres where the specimen was found
eventRemarks	Notes about the event
Table of Species Occurrence	Table with species occurrence data (beginning of new table)
occurrenceID	Identifier of the record, coded as a global unique identifier
institutionID	The identifier for the institution having custody of the object or information referred to in the record
institutionCode	The acronym of the institution having custody of the object or information referred to in the record
collectionID	An identifier of the collection to which the record belongs
collectionCode	The name of the collection from which the record was derived
datasetName	The name identifying the dataset from which the record was derived
eventID	Identifier of the event, unique for the dataset
kingdom	Kingdom name
phylum	Phylum name
class	Class name
order	Order name
family	Family name
genus	Genus name
specificEpithet	The name of the first or species epithet of the scientificName
infraspecificEpithet	The name of the lowest or terminal infraspecific epithet of the scientificName, excluding any rank designation
acceptedNameUsage	The specimen accepted name, with authorship
previousIdentifications	Previous name of the specimen, with authorship
scientificName	The name without authorship applied on the first identification of the specimen
basisOfRecord	The specific nature of the data record
habitat	Description of the habitat where the specimen was found
organismQuantityType	The type of quantification system used to quantity the organisms
organismQuantity	Percentage of the organism coverage
recordedBy	Person(s) responsible for sampling
catalogNumber	Identifying code for a unique sample lot in a biological collection
identifiedBy	Person(s) responsible for taxa identification
type	The nature of the resource
preparations	The preservation method used for the specimen
establishmentMeans	The establishment status of the organism in the study region
occurrenceRemarks	New record status assignment
licence	Reference to the licence under which the record is published

## Additional information

This paper accommodates the 418 specimens of macroalgae recorded from Terceira Island in 147 taxa (Tables 2, 3) comprising 113 confirmed species and 34 taxa identified only to genus level, belonging to 21 orders and 45 families, distributed by the phyla Rhodophyta (9 orders and 25 families), Chlorophyta (5 orders and 8 families) and Ochrophyta (7 orders and 12 families). The confirmed species include 73 Rhodophyta, 24 Chlorophyta and 16 Ochrophyta (Phaeophyceae). From these, 35 species are newly-recorded for the Island (27 Rhodophyta, 6 Chlorophyta and 2 Ochrophyta). Most species are native, including the three Macaronesian endemics *Millerella tinerfensis* (Seoane-Camba) S.M.Boo & J.M.Rico, *Phyllophora gelidioides* P.Crouan & H.Crouan ex Karsakoff and *Codium elisabethiae* O.C. Schmidt, eight are introduced and 15 have uncertain origin.

Many species were only sporadically recorded on Terceira, but nine were commonly found around the island and occurred quite abundantly in some locations, namely: the Rhodophyta *Asparagopsis armata* Harvey, *Ellisolandia elongata* and *Pterocladiella capillacea* (S.G. Gmelin) Santelices & Hommersand; the Chlorophyta *Ulva rigida* and *Ulva compressa* Linnaeus; and the Ochrophyta *Colpomenia sinuosa* (Mertens ex Roth) Derbès & Solier in Castagne, *Halopteris filicina* (Grateloup) Kützing, *Halopteris scoparia* (Linnaeus) Sauvageau and *Zonaria tournefortii*.

A mismatch regarding the GBIF backbone taxonomy of some of the macroalgae species names was identified as detailed in Suppl. material 1.

## Supplementary Material

2F0F41CA-5BC5-5743-BCB3-76FB77ADCFA0 10.3897/BDJ.8.e57462.suppl1 Supplementary material 1 DP-TER-id_14160_normalized-redz.csv Data type Macroalgae taxonomic mismatching Brief description GBIF does not have the more actualised nomenclature for some of the macroalgae species names. Therefore, the matching tools of its platform were applied to the species list, as required by Pensoft's data auditor, to identify the problematic taxonomic situations. The resulting file (DP-TER-id_14160_normalized-redz.csv) is included here, since the names will not be immediately updated in the GBIF Taxonomic Backbone. A request was already sent to GBIF helpdesk to resolve this situation. File: oo_438335.csv https://binary.pensoft.net/file/438335 Ana I Neto

## Figures and Tables

**Table 1.** Terceira Island sampling sites information.

Location No	Location ID	Municipality	Locality	Latitude / Longitude	geodeticDatum	Littoral zone
1	TER_AH_PJse	Angra do Herísmo	Porto Judeu|Serretinha	38,64491, -27,143929	WGS84	Intertidal
2	TER_AH_SSim	Angra do Herísmo	São Sebastião|lhéu da Mina	38,648825, -27,07385	WGS84	Intertidal
3	TER_PV_Bpi	Praia da Vitória	Biscoitos|Piscina	38,801473, -27,25893	WGS84	Intertidal
4	TER_AH_CRem	Angra do Herísmo	Cinco Ribeiras|Entre-marés	38,675345, -27,329175	WGS84	Intertidal
5	TER_AH_CR30	Angra do Herísmo	Cinco Ribeiras|30m	38.672771, -27.330059	WGS84	Subtidal
6	TER_AH_CRb	Angra do Herísmo	Cinco Ribeiras|Baía	38.675145, -27.327739	WGS84	Subtidal
7	TER_AH_CRem	Angra do Herísmo	Cinco Ribeiras|Entre-marés	38,675419, -27,329237	WGS84	Intertidal
8	TER_AH_Sb	Angra do Herísmo	Salga|Baía	38.645312, -27.097203	WGS84	Subtidal
9	TER_AH_Sem	Angra do Herísmo	Salga|Entre-marés	38,646749, -27.099061	WGS84	Intertidal
10	TER_AH_SIb	Angra do Herísmo	Silveira|Baía	38.653707, -27.233297	WGS84	Subtidal
11	TER_AH_SIem	Angra do Herísmo	Silveira|Entre-marés	38,655274, -27.237065	WGS84	Intertidal
12	TER_PV_Bb	Praia da Vitória	Biscoitos|Baía	38.800878, -27.260303	WGS84	Subtidal
13	TER_PV_Bpi	Praia da Vitória	Biscoitos|Piscina	38,801473, -27,25893	WGS84	Intertidal
14	TER_PV_Bpo	Praia da Vitória	Biscoitos|Ponta	38.804734, -27.255472	WGS84	Subtidal
15	TER_PV_PVb	Praia da Vitória	Praia da Vitória|Baía	38.7088, -27.048377	WGS84	Subtidal
16	TER_PV_PVem	Praia da Vitória	Praia da Vitória|Entre-marés	38.707052, -27.046829	WGS84	Intertidal
17	TER_PV_PVp	Praia da Vitória	Praia da Vitória|Paul	38,735015, -27,060895	WGS84	Intertidal
18	TER_PV_PVp	Praia da Vitória	Praia da Vitória|Paul	38,735015, -27,060895	WGS84	Intertidal

**Table 2.** Macroalgae species from Terceira Island, with information on their relative abundance, origin and status.

**Phylum**	**Species (Accepted Name)**	**Number of records**	**Establishment Means**	**Occurrence** **Remarks**
Rhodophyta	*Acrosorium ciliolatum* (Harvey) Kylin	4	Native	New record
Rhodophyta	*Amphiroa beauvoisii* J.V.Lamouroux	1	Native	
Rhodophyta	*Amphiroa fragilissima* (Linnaeus) J.V.Lamouroux	2	Native	New record
Rhodophyta	*Amphiroa rigida* J.V.Lamouroux	1	Native	New record
Rhodophyta	*Anotrichium tenue* (C.Agardh) Nägeli	2	Native	
Rhodophyta	*Aphanocladia stichidiosa* (Funk) Ardré	4	Native	
Rhodophyta	*Asparagopsis armata* Harvey	12	Introduced	
Rhodophyta	*Asparagopsis armata* Harvey, phase *Falkenbergia rufolanosa* (Harvey) F.Schmitz	1	Introduced	New record
Rhodophyta	*Asparagopsis taxiformis* (Delile) Trevisan	5	Native	
Rhodophyta	*Bonnemaisonia hamifera* Hariot	1	Introduced	
Rhodophyta	*Carradoriella denudata* (Dillwyn) A.M.Savoie & G.W.Saunders	3	Uncertain	
Rhodophyta	*Caulacanthus ustulatus* (Mertens ex Turner) Kützing	5	Uncertain	
Rhodophyta	*Centroceras clavulatum* (C.Agardh) Montagne	4	Native	
Rhodophyta	*Ceramium ciliatum* (J.Ellis) Ducluzeau	3	Native	
Rhodophyta	*Ceramium cingulatum* Weber Bosse	1	Introduced	
Rhodophyta	*Ceramium diaphanum* (Lightfoot) Roth	5	Native	
Rhodophyta	*Ceramium echionotum* J.Agardh	2	Native	
Rhodophyta	*Ceramium tenerrimum* (G.Martens) Okamura	1	Native	New record
Rhodophyta	*Ceramium virgatum* Roth	5	Native	
Rhodophyta	*Chondracanthus acicularis* (Roth) Fredericq	5	Native	
Rhodophyta	*Chondracanthus teedei* (Mertens ex Roth) Kützing	2	Native	New record
Rhodophyta	*Chondria coerulescens* (J.Agardh) Sauvageau	1	Uncertain	
Rhodophyta	*Chondria dasyphylla* (Woodward) C.Agardh	3	Uncertain	
Rhodophyta	*Crouania attenuata* (C.Agardh) J.Agardh	3	Native	
Rhodophyta	*Dermocorynus dichotomus* (J.Agardh) Gargiulo, M.Morabito & Manghisi	2	Native	
Rhodophyta	*Ellisolandia elongata* (J.Ellis & Solander) K.R.Hind & G.W.Saunders	10	Native	
Rhodophyta	*Gastroclonium clavatum* (Roth) Ardissone	4	Native	
Rhodophyta	*Gastroclonium ovatum* (Hudson) Papenfuss	1	Native	New record
Rhodophyta	*Gastroclonium reflexum* (Chauvin) Kützing	4	Native	
Rhodophyta	*Gayliella flaccida* (Harvey ex Kützing) T.O.Cho & L.J.McIvor	1	Native	New record
Rhodophyta	*Gelidium microdon* Kützing	7	Native	
Rhodophyta	*Gelidium pusillum* (Stackhouse) Le Jolis	5	Native	
Rhodophyta	*Gelidium spinosum* (S.G.Gmelin) P.C.Silva in Silva, Basson & Moe	5	Native	
Rhodophyta	*Gymnogongrus crenulatus* (Turner) J.Agardh	5	Native	
Rhodophyta	*Gymnogongrus griffithsiae* (Turner) C.Martius	3	Native	
Rhodophyta	Herposiphonia secunda f. secunda (C.Agardh) Falkenberg	4	Native	
Rhodophyta	*Hypnea arbuscula* P.J.L.Dangeard	1	Native	New record
Rhodophyta	*Hypnea musciformis* (Wulfen) J.V.Lamouroux	4	Uncertain	
Rhodophyta	*Jania capillacea* Harvey	4	Native	New record
Rhodophyta	*Jania longifurca* Zanardini	1	Uncertain	
Rhodophyta	Jania pedunculata var. adhaerens (J.V.Lamouroux) A.S.Harvey, Woelkerling & Reviers	3	Native	New record
Rhodophyta	*Jania pumila* J.V.Lamouroux	1	Native	New record
Rhodophyta	*Jania rubens* (Linnaeus) J.V.Lamouroux	3	Native	
Rhodophyta	*Jania virgata* (Zanardini) Montagne	3	Uncertain	
Rhodophyta	*Laurencia chondrioides* Børgesen	1	Introduced	
Rhodophyta	*Laurencia minuta* Vandermeulen, Garbary & Guiry	2	Introduced	New record
Rhodophyta	*Laurencia tenera* C.K.Tseng	3	Native	New record
Rhodophyta	*Lomentaria articulata* (Hudson) Lyngbye	4	Native	
Rhodophyta	*Lomentaria clavellosa* (Lightfoot ex Turner) Gaillon	1	Uncertain	
Rhodophyta	*Lomentaria orcadensis* (Harvey) Collins in W.R.Taylor	1	Uncertain	
Rhodophyta	*Lophosiphonia cristata* Falkenberg	5	Native	New record
Rhodophyta	*Melanothamnus sphaerocarpus* (Børgesen) Díaz-Tapia & Maggs	2	Introduced	
Rhodophyta	*Meredithia microphylla* (J.Agardh) J.Agardh	2	Native	New record
Rhodophyta	*Millerella pannosa* (Feldmann) G.H.Boo & L.Le Gall	2	Native	New record
Rhodophyta	*Millerella tinerfensis* (Seoane-Camba) S.M.Boo & J.M.Rico	3	Macaronesian endemism	New record
Rhodophyta	*Nitophyllum punctatum* (Stackhouse) Greville	1	Native	
Rhodophyta	*Osmundea hybrida* (A.P.de Candolle) K.W.Nam	1	Native	New record
Rhodophyta	*Osmundea pinnatifida* (Hudson) Stackhouse	6	Native	
Rhodophyta	*Osmundea truncata* (Kützing) K.W.Nam & Maggs in K.W.Nam, Maggs & Garbary	4	Native	New record
Rhodophyta	*Peyssonnelia squamaria* (S.G.Gmelin) Decaisne ex J.Agardh	1	Native	
Rhodophyta	*Phyllophora gelidioides* P.Crouan & H.Crouan ex Karsakoff	2	Native	New record
Rhodophyta	*Plocamium cartilagineum* (Linnaeus) P.S.Dixon	3	Native	
Rhodophyta	*Pterocladiella capillacea* (S.G.Gmelin) Santelices & Hommersand	9	Native	
Rhodophyta	*Rhodophyllis divaricata* (Stackhouse) Papenfuss	4	Native	New record
Rhodophyta	*Rhodymenia holmesii* Ardissone	5	Native	New record
Rhodophyta	*Sphaerococcus coronopifolius* Stackhouse	1	Native	New record
Rhodophyta	*Sphondylothamnion multifidum* (Hudson) Nägeli	2	Native	
Rhodophyta	*Spyridia filamentosa* (Wulfen) Harvey	2	Native	New record
Rhodophyta	*Symphyocladia marchantioides* (Harvey) Falkenberg	2	Introduced	
Rhodophyta	*Vertebrata fruticulosa* (Wulfen) Kuntze	1	Native	New record
Rhodophyta	*Vertebrata hypnoides* (Welwitsch) Kuntze	2	Uncertain	
Rhodophyta	*Vertebrata reptabunda* (Suhr) Díaz-Tapia & Maggs	4	Uncertain	
Rhodophyta	*Vertebrata tripinnata* (Harvey) Kuntze	1	Native	
Rhodophyta	*Wurdemannia miniata* (Sprengel) Feldmann & Hamel	2	Native	New record
Chlorophyta	*Blidingia minima* (Nägeli ex Kützing) Kylin	1	Native	New record
Chlorophyta	*Bryopsis cupressina* J.V.Lamouroux	1	Native	New record
Chlorophyta	*Bryopsis plumosa* (Hudson) C.Agardh	3	Native	
Chlorophyta	*Chaetomorpha aerea* (Dillwyn) Kützing	5	Native	
Chlorophyta	*Chaetomorpha linum* (O.F.Müller) Kützing	1	Native	
Chlorophyta	*Chaetomorpha mediterranea* (Kützing) Kützing	1	Native	New record
Chlorophyta	*Chaetomorpha pachynema* (Montagne) Kützing	2	Native	
Chlorophyta	*Cladophora albida* (Nees) Kützing	2	Native	
Chlorophyta	*Cladophora coelothrix* Kützing	5	Native	
Chlorophyta	*Cladophora dalmatica* Kützing	1	Uncertain	
Chlorophyta	*Cladophora laetevirens* (Dillwyn) Kützing	2	Uncertain	
Chlorophyta	*Cladophora lehmanniana* (Lindenberg) Kützing	1	Native	New record
Chlorophyta	*Cladophora prolifera* (Roth) Kützing	5	Native	
Chlorophyta	*Cladophoropsis membranacea* (Hofman Bang ex C.Agardh) Børgesen	1	Uncertain	
Chlorophyta	*Codium adhaerens* C.Agardh	4	Native	
Chlorophyta	*Codium elisabethiae* O.C.Schmidt	1	Macaronesian endemism	
Chlorophyta	*Gayralia oxysperma* (Kützing) K.L.Vinogradova ex Scagel	1	Native	New record
Chlorophyta	*Lychaete pellucida* (Hudson) M.J.Wynne	3	Native	New record
Chlorophyta	*Ulva clathrata* (Roth) C.Agardh	2	Native	
Chlorophyta	*Ulva compressa* Linnaeus	6	Native	
Chlorophyta	*Ulva intestinalis* Linnaeus	5	Native	
Chlorophyta	*Ulva polyclada* Kraft	1	Native	
Chlorophyta	*Ulva prolifera* O.F.Müller	5	Native	
Chlorophyta	*Ulva rigida* C.Agardh	6	Native	
Ochrophyta	*Asterocladon rhodochortonoides* (Børgesen) S.Uwai, C.Nagasato, T.Motomura & K.Kogame	1	Native	
Ochrophyta	*Cladostephus spongiosus* (Hudson) C.Agardh	1	Native	
Ochrophyta	*Colpomenia sinuosa* (Mertens ex Roth) Derbès & Solier	10	Native	
Ochrophyta	*Dictyota dichotoma* (Hudson) J.V.Lamouroux	1	Native	
Ochrophyta	*Feldmannia irregularis* (Kützing) Hamel	1	Native	
Ochrophyta	*Fucus spiralis* Linnaeus	5	Uncertain	
Ochrophyta	*Halopteris filicina* (Grateloup) Kützing	13	Native	
Ochrophyta	*Halopteris scoparia* (Linnaeus) Sauvageau	12	Native	
Ochrophyta	*Nemoderma tingitanum* Schousboe ex Bornet	5	Native	
Ochrophyta	*Padina pavonica* (Linnaeus) Thivy	4	Native	
Ochrophyta	*Petalonia binghamiae* (J.Agardh) K.L.Vinogradova	1	Introduced	
Ochrophyta	*Pseudolithoderma adriaticum* (Hauck) Verlaque	2	Native	New record
Ochrophyta	*Ralfsia verrucosa* (Areschoug) Areschoug	7	Native	
Ochrophyta	*Sargassum cymosum* C.Agardh	1	Native	New record
Ochrophyta	*Treptacantha abies-marina* (S.G.Gmelin) Kützing	4	Native	
Ochrophyta	*Zonaria tournefortii* (J.V.Lamouroux) Montagne	8	Native	

**Table 3.** Main taxonomic figures with information on the species origin and status.

**Phyllum**	**Order**	**Family**	**Specimens Number**	**Total taxa**	**Total species**	**Native**	**Introduced**	**Uncertain**	**Macaronesian endemism**	**New record**
Rhodophyta	9	25	248	95	73	53	7	11	2	27
Chlorophyta	5	8	77	33	24	20		3	1	6
Ochrophyta	7	12	93	19	16	14	1	1		2
**Total**	**21**	**45**	**418**	**147**	**113**	**87**	**8**	**15**	**3**	**35**
